# Roasting treatments affect oil extraction rate, fatty acids, oxidative stability, antioxidant activity, and flavor of walnut oil

**DOI:** 10.3389/fnut.2022.1077081

**Published:** 2023-01-04

**Authors:** Huankang Li, Jiajia Han, Zhongkai Zhao, Jinhu Tian, Xizhe Fu, Yue Zhao, Changqing Wei, Wenyu Liu

**Affiliations:** ^1^School of Food Science and Technology, Shihezi University, Shihezi, China; ^2^College of Life Sciences and Technology, Xinjiang University, Ürümqi, China; ^3^College of Biosystems Engineering and Food Science, Zhejiang University, Hangzhou, China; ^4^Key Laboratory of Xinjiang Phytomedicine Resource and Utilization of Ministry of Education, Shihezi University, Shihezi, China

**Keywords:** walnut oil, roasting treatment, physicochemical characteristics, antioxidant capacity, correlation analysis, volatile organic compounds, HS-GC-IMS

## Abstract

**Introduction:**

The quality of pressed walnut oil can be improved by moderate roasting treatment.

**Methods:**

This study compared physicochemical characteristics and antioxidant ability of walnut oils pressed from differently roasted pretreated walnuts, analyzed the correlation among these indicators by using Pearson correlation coefficient and correlation coefficient heatmap, and evaluated the volatile organic compounds (VOCs) of walnut oil under optimal pretreatment roasting conditions using headspace-gas chromatography-ion mobility spectrometry (HS-GC-IMS).

**Results:**

Hierarchical cluster analysis (HCA) and principal component analysis (PCA) were able to remarkably distinguish walnut oil produced by different roasting processes. In addition, correlation analysis showed that there was a significant impact among indicators. There were 73 VOCs were identified in the optimum roasted treated walnut oil, consisting of 30 aldehydes, 13 alcohols, 11 ketones, 10 esters, 5 acids, 2 oxygen-containing heterocycles, 1 nitrogen-containing heterocycle and 1 other compound. GC-IMS results showed that aldehydes contributed significantly to the volatile flavor profile of walnut oil, especially (E)-2-heptenal, (E)-2-pentenal and hexenal.

**Discussion:**

The properties of walnut oil based on varying roasting pretreatment of walnut kernels were significantly differentiated. Roasting at 120°C for 20 min is a suitable pretreatment roasting condition for pressing walnut oil. Roasting at 120°C for 20 min is a suitable pretreatment roasting condition for pressing walnut oil.

## Graphical Abstract

The physicochemical properties and antioxidant capacity of walnut oil obtained by pressing walnut kernels after different roasting times and temperatures were compared, and the correlations between these indicators were analyzed using Pearson correlation coefficients and correlation coefficient heat maps, and the volatile organic compounds of walnut oil under optimal pretreatment roasting conditions were evaluated using headspace-gas chromatography-ion mobility spectrometry. 
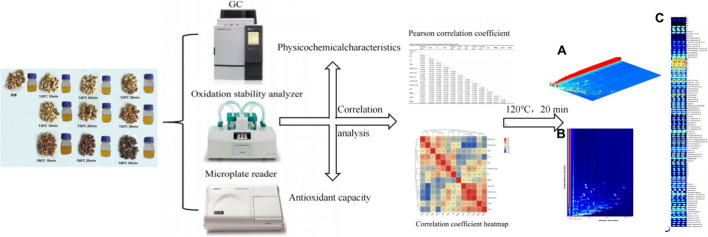

## 1. Introduction

Walnut is a perennial deciduous tree of the genus Juglans, and with almonds, cashews and hazelnuts are known as the four dried fruits. At present, there are 21 species of walnut, which are widespread in the West Indies, southern Europe, Asia, Central America, North America, and western South America (1). The global walnut cultivation area reaches about 1.3 million hectares. Turkey, the United States, and China are the three major walnut producing countries, with the combined area and production of walnuts accounting for 69.22 and 81.32% of the world total, respectively (2). The main production areas of walnut in China include Yunnan Province (880,000 tons), Xinjiang Uygur Autonomous Region (440,000 tons), Sichuan Province (300,000 tons), Shaanxi Province (200,000 tons), Hebei Province (120,000 tons) and Guizhou Province (90,000 tons). Xinjiang’s unique climatic conditions have created the excellent quality of Xinjiang walnuts. Hetian, Kashi, and Aksu are the three major walnut producing areas in Xinjiang of China, which main varieties include Zha No. 343, Xinfeng, Wen 185, Xinzaofeng, Xinxin No. 2. Walnuts contain large amounts of monounsaturated fatty acids (MUFAs) and polyunsaturated fatty acids (PUFAs), with an oil content of 60–70% (3). The ratio of ω-6: ω-3 PUFAs in walnut oil is generally 4:1, which is in line with the optimal ratio proposed by the World Health Organization (WHO) and the Food and Agriculture Organization of the United Nations (FAO). The flavonoids in walnut oil can relieve headache, tinnitus, dizziness, and other symptoms. The rich phospholipids in walnut oil can enhance cell activity, promote hematopoiesis, and protect brain nerves. Walnut oil is also rich in vitamin E, which plays an important role in scavenging free radicals and delaying aging (4, 5). However, the high content of unsaturated fatty acids in walnut oil can make it prone to oxidation, resulting in a short shelf life (6). In recent years, with the increasing interest in fats and oils from a health and nutritional perspective, consumers have become increasingly interested in unrefined vegetable oils, especially cold-pressed obtained (7). Cold pressing is the use of mechanical force to produce oil at a temperature below 60°C, such as poppy seed oil, pumpkin oil and rapeseed oil (8–12). Additionally, roasting is a pretreatment method for nut kernels before the oil extraction, which can effectively improve the flavor in the oil (13). Antioxidant activity and phenolic content of nuts such as peanuts, hazelnuts and cashews as affected by roasting treatment (14, 15). Chandrasekara and Shahidi treated the cashew at low and high temperatures, respectively, which confirmed that with the increase of roasting temperature, their antioxidant activity would also increase (16). The highest activity was achieved when nuts were roasted at 130°C for 33 min. This implied that the antioxidant effect of processed foods would be enhanced by Maillard reaction products generated during heat treatment (17). In addition, the fatty acid composition of walnut and hazelnut changed after the roasting treatment (18, 19). Lin et al. researched show that roasting enhanced the content of linoleic acid, oleic acid, and linolenic acid (20). In summary, moderate roasting may also cause changes in the physicochemical properties and antioxidant capacity of walnut oil. However, most of the researches focus on the change of properties caused by the roasting process, but the correlation between these properties has been insufficiently studied. Therefore, this study aimed to investigate and elucidate the correlation between the antioxidant capacity and physicochemical properties of walnut oil.

Odor is one of the typical characteristics of vegetable oil flavor, which is an important index to judge the quality of plant oil. It is an important factor in affecting consumer choice. The flavor of plant oils depends on the variety, ripeness degree, environmental condition, growing region, storage, and processing techniques (21–23). Processing technique can significantly affect the concentrations of major volatile components, and thus causing different flavors in plant oils (24). Headspace gas chromatography-ion mobility spectrometry (HS-GC-IMS) is a recent instrumentation technique for the separation and detection of volatile organic compounds (VOCs) in mixed analytes (25). GC is widely applied to the measurement of volatiles. IMS has been applied mainly in airport security and military applications in the quick identification of chemical narcotics, explosives and warfare agents owing to its high sensitivity (26). Simultaneously, IMS has also been demonstrated valuable in other applications, like interior and exterior environmental quality control, food and pharmaceutical quality control, and respiratory disease monitoring (27–29). Specifically, IMS is developing extremely well in the food industry to examine information concerning food origin, food raw materials, food quality and food aroma (30–33). Therefore, HS-GC-IMS was utilized for the rapid identification of VOCs in walnut oil obtained from the suitable pretreatment roasting conditions determined in this research.

Pretreatment of walnut kernels through different roasting temperatures and durations will affect the physicochemical characteristics, antioxidant capacity and flavor of walnut oil. Walnut oil was prepared by the cold pressing method, using walnut kernels at various roasting temperatures (120, 150, and 180°C) and different durations (10, 20, and 30 min) in this paper. The fatty acid, physicochemical characteristics, free radical scavenging capacity and oxidative stability index (OSI) of the oils were investigated. Then, correlation analysis was performed on these indicators by the Pearson correlation coefficient and the correlation coefficient heatmap. HS-GC-IMS was utilized for the rapid identification of VOCs in walnut oil obtained from the determined suitable pretreatment roasting conditions. These results have significant implications for improving research and guiding the market.

## 2. Materials and methods

### 2.1. Walnut samples collection

The walnut samples were the Wen 185 walnuts from Aletai, Xinjiang, China. These walnuts were picked up in the Xinjiang Autonomous Region in 2019–2020.

### 2.2. Chemicals

Palmitic, stearic, oleic, linoleic, and linolenic acid standards, 2,2-Diphenyl-1-picrylhydrazyl (DPPH), 2,2′-Azino-bis (3-ethylbenzothiazoline-6-sulfonic acid) diammonium salt (ABTS) and 2,4,6-Tris (2-pyridyl)-1,3,5-triazine (TPTZ) were purchased from Sigma-Aldrich Inc. (St. Louis, MO). Sigma-Aldrich Chemical Co., Ltd. (Shanghai, China). Additional solvents and reagents were purchased from Tianjin Fuyu Fine Chemical Co. (Tianjin, China).

### 2.3. Walnut oil preparation

#### 2.3.1. Roasting temperature and duration

The walnut kernels were divided at random into 30 groups of 300 g for each part, of which 27 portions were applied in roasting, and the remaining 3 were used as blank controls. The roasting temperature was set to 120, 150, and 180°C, and the roasting durations was set to 10, 20, and 30 min. A total of nine different groups of roasting treatments were carried out, with three parallel treatments in each group. Each sample is baked in the electric oven (K1H, Guangdong Galanz Microwave and Electrical Appliances Manufacturing Co., Ltd., China). The roasted walnuts were cooled to ambient temperature, stored in a sealed plastic bag, and placed in a 4°C refrigerator pending the next stage of experimentation.

#### 2.3.2. Sample preparation

Walnut kernels were transferred to a screw press (ZY-22A, Wenzhou Hongkuo Technology Co., Ltd., China). The temperature in the press is 30°C, and the oil output temperature was 30 ± 2°C. The turbid oil was centrifuged at 8,000 r/min for 20 min, and then the supernatant oil was directly transferred to a 100 mL dark bottle for sealing storage, pending further experiments.

### 2.4. Determination of fatty acid

#### 2.4.1. Fatty acid methyl esterification

The fatty acid methyl esterification (FAME) preparation followed the prior procedure except for some modifications (20). The crude walnut oil (200 mg) with 8 mL of 2% NaOH (prepared with methanol) refluxed in a water bath at 80 ± 1°C until the oil droplets disappeared, then 7 mL 15% BF_3_ (made by methanol) was added to the conical flask and reacted for 2 min to completely derivatize the methyl ester. For the purification of methyl ester, 20 mL of hexane and 3 mL of saturated NaCl solution were added. The hexane extracted FAME products were dried with anhydrous Na_2_SO_4_ and then filtered through a 0.22 μm filter before being analyzed by GC.

#### 2.4.2. GC analysis

The fatty acid was analyzed using a GC-2014 gas chromatograph (Shimadzu, Kyoto, Japan) and a HP-INNOWAX capillary column (0.25 μm, 30 m × 0.25 mm, Agilent, USA).

The operating conditions were as follows: nitrogen with a linear velocity of 1.0 mL/min as carrier gas, the flame ionization detector (FID) (Thermo Fisher), the sampler temperature was 240°C, injection mode was no split, injection volume was 1.0 μL, the chromatographic column adopted a gradient temperature increase, kept at 140°C for 2 min, then increased the temperature to 180°C at 5°C/min and held for 5 min, and finally increased to 230°C at 5°C/min to maintain 6 min. The fatty acid composition was analyzed by comparing the retention time on the chromatogram of the sample and the FAME standard, and the fatty acid content was expressed as a relative percentage.

### 2.5. Determination of physicochemical properties

#### 2.5.1. Determination of peroxide value

A mixture of 2 mL of oil sample, 1 mL of saturated potassium iodide solution and 30 mL of chloroform-glacial acetic acid (2:3, v/v) was added sequentially to a 250 mL conical flask, shaken for 0.5 min and allowed to stand for 3 min. Subsequently, 100 mL of distilled water was added to the conical flask and the mixture was titrated to light yellow with 0.001 mol/L of Na_2_S_2_O_3_⋅5H_2_O, 1 mL of starch indicator was added and the titration was continued until the blue color of the solution disappeared.

#### 2.5.2. Determination of acid value

The mixture of 50 mL 95% ethanol and 0.5 mL phenolphthalein indicator was heated to a slight boil in a 95°C water bath and titrated to red in 0.1 mol/L NaOH solution while hot. Twenty grams of oil sample was added, and the reaction was continued to be heated to a slight boil in a 95°C water bath and titrated to a slight red color.

#### 2.5.3. Determination of 2-thiobarbituric acid

The thiobarbituric acid (TBA) reagent was 200 mg of 2-TBA with 1-butanol to 100 mL volumetric flask. After 12–15 h at room temperature, it was filtered and the filtrate was placed in a refrigerator at 4°C for use. After the 200 mg of oil sample was made up to 25 mL with 1-butanol, 5 mL of the mixture was mixed with TBA regent and allowed to stand in a water bath at 95°C for 2 h. The absorbance was measured at 530 nm with the spectrophotometer (T600, Beijing Puxi General Instrument Co., Ltd., Beijing, China).

### 2.6. Determination of antioxidant capacity

#### 2.6.1. DPPH radical assay

The measurement was performed based on a modified reported method (34). Dissolve 5 g of walnut oil in 20 mL of methanol and mix, keep shaking and extract for 30 min at 50°C, centrifuge at low temperature (8,000 r/min) for 5 min. The supernatant was aspirated and the extraction process was repeated 4 times and the combined supernatant was polarized. Mix 1 mL of the polar substances with 1 mL of 1 mM DPPH (prepared with methanol), react in the dark at room temperature for 30 min. The absorbance of the reaction solution and the blank were measured at 517 nm. Meanwhile, different concentrations of Vitamin E (V_E_) solutions were used instead of the sample as a control test. The results were showed as the equivalent of V_E_.

#### 2.6.2. Ferric reducing antioxidant power assay

The Ferric reducing antioxidant power (FRAP) method was performed according to the previous procedure with some modifications (35). The extraction of polar components of walnut oil was the same as that of DPPH. FRAP reagent was prepared by mixing 20 mmol/L FeCl_3_⋅6H_2_O solution, 10 mmol/L TPTZ solution (40 mmol/L HCl), and 0.1 mol/L sodium acetate buffer solution (pH 3.6) in a ratio of 1:1:10. The oil polar substance (200 μL) was dissolved into FRAP reagent (2 mL) and fixed to 10 mL with deionized water and reacted at 37°C for 10 min. Dissolve 27.802 mg FeSO_4_⋅7H_2_O into 100 mL with distilled water to configure 1 mmol/L FeSO_4_ solution. After dilution, different concentration gradients of FeSO_4_ and FRAP reagent were reacted to obtain FeSO_4_ standard curve. The absorbance of the reaction solution and the blank were measured at 593 nm. Meanwhile, different concentrations of V_E_ solutions were used as control tests instead of the samples, and the results were expressed as the equivalent of V_E_.

#### 2.6.3. ABTS radical assay

ABTS was determined using the reported method with slight modifications (36). The extraction of polar components of walnut oil was the same as the extraction method in DPPH. The ABTS working solution was prepared by mixing 7 mmol/L ABTS and 2.45 mmol/L potassium persulfate solution in equal amounts, and placed in the dark at room temperature for 12–16 h. The ABTS working solution was diluted with ethanol to an absorbance of 0.700 ± 0.020 at 734 nm for analysis. The 200 μL of oil polar components and 4 mL of ABTS radical solution were mixed in the dark at room temperature for 20 min. The absorbance of the reaction solution and the blank were measured at 734 nm. Meanwhile, different concentrations of V_E_ solutions were used as control tests instead of the samples, and the results were expressed as the equivalent of V_E_.

#### 2.6.4. Oxidative stability index

The OSI was measured by the 892 professional Rancimat oil oxidation stability analyzer (Metrohm China Co., Ltd., Beijing, China) at 110°C and 20 L/h airflow. Use 5 g walnut oil for the measurement. High temperature and excessive air can accelerate the oxidation of glycerol fatty acid esters and produce volatile organic acids. The air brought volatile organic acids into a conductive chamber and changed the conductivity of water. The time period before the conductivity increases sharply (OSI time) was measured by continuously measuring the conductivity of the conductive chamber.

### 2.7. HS-GC-IMS analysis

The GC-IMS instrument (FlavourSpec^®^, the G.A.S. Department of Shandong Hai Neng Science Instrument Co., Ltd., Shandong, China) is equipped with a syringe and an automatic headspace research sampler unit. The sample entered the instrument with the carrier gas, passed through the gas chromatographic column for the initial separation, and then entered the ion transfer tube. Through the action of the reverse drift gas, it migrated to the Faraday disc for detection and realized the secondary separation.

In this study, 2.0 g sample of walnut oil sample was accurately weighed into a 20 mL headspace glass sampling vial and incubated at 500 rpm, 80°C for 20 min. Subsequently, 200 μL of headspace was automatically injected into the injector (85°C) Separation of VOCs was performed by the gas chromatographic column MXT-5 capillary column (15 m × 0.53 mm, 60°C) and the carrier gas consisted of 99.99% pure nitrogen. The programmed nitrogen flow rate was: 2 mL/min for 2 min; increased to 100 mL/min within the 2–20 min. The drift tube was maintained at 45°C under N_2_ as a drift gas at 150 mL/min. the total running time was 30 min.

Retention indices (National Institute of Standards and Technology database) and drift times (IMS database) were used to perform qualitative analysis of VOCs in the sample.

### 2.8. Statistical analysis

Statistical analyses were carried out using Unscrambler version 9.7 (CAMO ASA, Oslo, Norway), Origin 2018 Pro (Originlab, Northampton, MA, USA) and SPSS 25.0 (IBM, Armonk, NY, USA). Correlation analysis was performed out by Pearson correlation coefficient and *t*-test. Chemometrics [principal component analysis (PCA) and Hierarchical cluster analysis (HCA)] were used to analyze the obtained data. The samples were determined three times and the results were recorded as mean ± standard deviation.

## 3. Results and discussion

### 3.1. Effect of roasting on oil extraction rate and fatty acid composition of walnut oil

**FIGURE 1:**
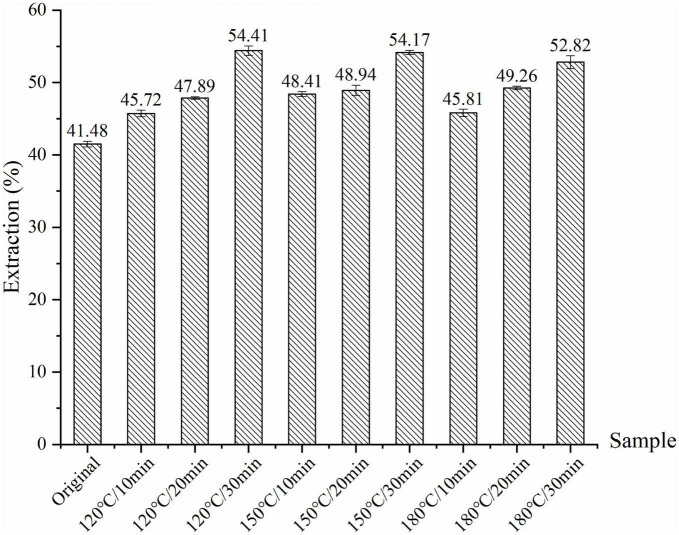
Extraction yields for walnut kernels roasted with different temperature and duration.

The crude oil yields ranged from 41.48 to 54.41% from roasted walnut kernels. The oil yield of the roasted sample was significantly higher than that of the unroasted sample (41.48%) (Figure 1). The roasting treatment caused a series of changes in the walnut kernels, including destroying oil cells, promoting protein denaturation, reducing oil viscosity, which were suitable for squeezing oil and increasing oil yield. The oil extraction yield of walnut kernels increases with the extension of roasting time. The highest oil yield was obtained for the sample roasted at 120°C for 30 min compared to the other samples.

Table 1 shows the fatty acid compositions of walnut oil pressed from roasted walnut kernels under different roasting conditions. In all samples studied, the main fatty acids identified were linoleic acid (C18:2) (50.74–62.65%), oleic acid (C18:1) (13.33–20.88%), linolenic acid (C18:3) (12.39–17.59%), palmitic acid (C16:0) (7.08–9.41%), and stearic acid (C18:0) (1.22–2.44%). PUFAs were the primary fatty acids, with 68.33–77.06%, and the second were MUFAs, with 13.33–20.88%. Linoleic acid was the main fatty acid in walnut oil. The lowest content of linoleic acid was found in samples baked at 120°C for 30 min, and the highest content of linoleic acid was found in walnut oil baked at 150°C for 30 min.

**TABLE 1:** Fatty acid composition (%) of walnut oil by roasted walnut kernels with different temperature and duration.

Sample		Palmitic acid	Stearic acid	Oleic acid	Linoleic acid	Linolenic acid	SFA	MUFA	PUFA
Original		7.08 ± 0.08^c^	2.43 ± 0.03^a^	15.79 ± 0.08^bc^	62.31 ± 0.11^a^	12.40 ± 0.08^d^	9.51 ± 0.12^cd^	15.79 ± 0.08^bc^	74.71 ± 0.19^ab^
120°C	10 min	7.99 ± 0.13^bc^	1.26 ± 0.25^c^	17.97 ± 0.05^ab^	58.87 ± 0.35^b^	13.94 ± 0.09^cd^	9.24 ± 0.38^d^	17.97 ± 0.05^ab^	72.80 ± 0.43^bc^
	20 min	8.70 ± 0.06^ab^	1.57 ± 0.02^bc^	19.02 ± 0.09^ab^	54.03 ± 0.45^c^	16.69 ± 0.40^ab^	10.28 ± 0.04^ab^	19.02 ± 0.09^ab^	70.72 ± 0.06^cd^
	30 min	9.41 ± 0.13^a^	1.39 ± 0.08^c^	20.88 ± 0.18^a^	50.74 ± 0.28^d^	17.59 ± 0.25^a^	10.80 ± 0.22^a^	20.88 ± 0.18^a^	68.33 ± 0.04^d^
150°C	10 min	8.84 ± 0.36^ab^	1.67 ± 0.01^abc^	18.73 ± 0.33^ab^	55.68 ± 0.70^c^	15.10 ± 0.74^bc^	10.51 ± 0.37^ab^	18.73 ± 0.33^ab^	70.77 ± 0.04^cd^
	20 min	7.41 ± 0.24^c^	1.22 ± 0.25^c^	16.28 ± 1.19^bc^	60.92 ± 2.09^a^	14.18 ± 0.91^cd^	8.63 ± 0.01^e^	16.28 ± 1.19^bc^	75.10 ± 1.18^ab^
	30 min	8.14 ± 1.19^bc^	1.89 ± 0.67^abc^	13.82 ± 3.01^c^	62.65 ± 1.07^a^	13.50 ± 1.43^cd^	10.02 ± 0.51^bc^	13.82 ± 3.01^c^	76.16 ± 2.50^a^
180°C	10 min	7.15 ± 0.11^c^	2.33 ± 0.01^ab^	15.91 ± 0.02^bc^	61.57 ± 0.06^a^	13.06 ± 0.02^cd^	9.48 ± 0.11^cd^	15.91 ± 0.02^bc^	74.62 ± 0.08^ab^
	20 min	7.82 ± 1.03^bc^	1.80 ± 0.72^abc^	13.33 ± 3.42^c^	62.46 ± 0.93^a^	14.61 ± 2.18^bcd^	9.61 ± 0.31^cd^	13.33 ± 3.42^c^	77.06 ± 3.11^a^
	30 min	7.10 ± 0.13^c^	2.44 ± 0.00^a^	16.62 ± 0.07^bc^	61.45 ± 0.07^a^	12.39 ± 0.01^d^	9.54 ± 0.13^cd^	16.62 ± 0.07^bc^	73.84 ± 0.06^abc^

Values (mean ± SD, *n* = 3) in the same column followed by a different letter are significantly different (*p* < 0.05).

The roasting treatment did not affect the composition of fatty acids in walnut oil, but the fatty acid content varied slightly after roasting. Moderate roasting can increase the content of unsaturated fatty acids (oleic acid and linoleic acid) and saturated fatty acids (palmitic acid), especially the sample roasted 120°C for 30 min. The results of Vaidya and Eun showed that there was no significant difference in the fatty acid composition of walnut oil obtained from roasted and unroasted walnut kernels, without considering the effect of roasting time and temperature (37). It is reported that the SFA content in perilla oil slightly increased after roasting treatment (38). Lin et al., reported that with the extension of temperature and duration, the content of unsaturated fatty acids and saturated fatty acids in almond kernel oil increased (20).

### 3.2. Physicochemical characteristics

Table 2 lists the physicochemical characteristics of the walnut oil. Walnut oil had a low acid value (AV) (0.12–0.37 mg NaOH/g), indicating that the roasting treatment did not promote the formation of free fatty acids in walnut oil. POV characterizes the degree of oil rancidity by measuring the oxidation products of the oil (4). The results revealed that the POV increased from 1.77 mmol/kg (120°C, 10 min) to 3.98 mmol/kg (180°C, 30 min) with increasing roasting temperature and time. Significant increase in POV indicated that the roasting treatment promoted the production of peroxides and hydroperoxides in walnut oil. However, the POV results still meet the standards for commercial edible vegetable oils (≤10 mmol/kg). TBA is directed to the oxidation products unsaturated fatty acid aldehydes, which are used to characterize the degree of oxidation in different oxidation stages. The resulting TBA values after roasting ranged from 0.0268 to 0.0318. Roasting at 120°C for 30 min, 150°C for 30 min and 180°C for 10 min had the lowest degree of oxidation at the corresponding temperature.

**TABLE 2:** Peroxide value, acid value, and thiobarbituric acid of walnut oil by roasted walnut kernels with different temperature and duration.

Sample		POV (mmol/kg)	AV (mg NaOH/g)	TBA
Original		1.59 ± 0.09^i^	0.12 ± 0.25^e^	0.0313 ± 0.0019^b^
120°C	10 min	1.77 ± 0.05^h^	0.23 ± 0.23^cd^	0.0315 ± 0.0006^b^
	20 min	1.93 ± 0.02^g^	0.23 ± 0.21^cd^	0.0318 ± 0.0005^b^
	30 min	2.00 ± 0.02^g^	0.20 ± 0.10^d^	0.0268 ± 0.0010^d^
150°C	10 min	2.34 ± 0.04^f^	0.29 ± 0.17^b^	0.0285 ± 0.0006^c^
	20 min	2.57 ± 0.06^e^	0.37 ± 0.10^a^	0.0303 ± 0.0010^b^
	30 min	2.71 ± 0.07^d^	0.21 ± 0.15^d^	0.0270 ± 0.0000^d^
180°C	10 min	3.58 ± 0.09^c^	0.19 ± 0.10^d^	0.0270 ± 0.0000^d^
	20 min	3.83 ± 0.05^b^	0.26 ± 0.31^bc^	0.0355 ± 0.0013^a^
	30 min	3.98 ± 0.12^a^	0.34 ± 0.53^a^	0.0318 ± 0.0009^b^

Values (mean ± SD, *n* = 3) in the same column followed by a different letter are significantly different (*p* < 0.05). POV (mmol/kg) = (volume consumed by the sample-volume consumed by the blank) × concentration of Na_2_S_2_O_3_⋅5H_2_O × 1,000/(2 × quality of oil sample). AV (mg NaOH/g) = volume consumed by the titrant × concentration of NaOH × 39.996/quality of oil sample. TBA = (50 × absorbance of the tested solution/sample weight) × 100%.

### 3.3. Analysis of antioxidant capacity

Table 3 lists the free radical scavenging ability (FRAP, ABTS, and DPPH) and OSI of walnut oil under different roasting conditions. The oxidation process of the walnut oil can be accelerated by exposing the walnut oil to 110°C and the air at a flow rate of 20 L/h (39). The oxidative stability of walnut oil was characterized by measuring the length of the induction time from the induction period to the oxidation period. The OSI of walnut oil ranged from 4.53 to 5.57 h, indicating that roasting caused increasing OSI of the oil. In the FRAP assay, the results ranged from 88.19 to 119.45 μmol TE/kg. For ABTS, the results were in the range of 14.76–95.48 μmol TE/kg. The DPPH results varied from 1.84 to 45.45 μmol TE/kg. Moderate roasting treatment could lead to a significant increase (*p* < 0.05) in the antioxidant activity of walnut oil. Roasting of kernels at 120°C for 20 min resulted in a significant increase (*p* < 0.05) in the ABTS and FRAP test values compared to those of others sample, while the highest value of DPPH appeared at 120°C for 10 min.

**TABLE 3:** Oxidative stability index (h) and free radical scavenging capacity (μmol TE/L) of walnut oil by roasted walnut kernels with different temperature and duration.

Sample		OSI	DPPH	ABTS	FRAP
Original		4.53 ± 0.31^c^	29.19 ± 3.21^b^	30.00 ± 0.72^d^	101.41 ± 2.86^c^
120°C	10 min	4.68 ± 0.12^c^	45.45 ± 2.37^a^	14.76 ± 0.42^h^	106.13 ± 1.61^b^
	20 min	5.34 ± 0.30^ab^	12.91 ± 1.57^d^	95.48 ± 0.83^a^	119.45 ± 1.22^a^
	30 min	4.77 ± 0.13^c^	18.56 ± 1.39^c^	26.44 ± 0.72^e^	102.31 ± 0.66^c^
150°C	10 min	4.78 ± 0.24^c^	13.59 ± 1.42^d^	21.44 ± 0.72^f^	109.07 ± 0.26^b^
	20 min	4.69 ± 0.23^c^	18.55 ± 1.55^c^	29.76 ± 0.83^d^	100.10 ± 1.46^cd^
	30 min	5.57 ± 0.20^a^	31.89 ± 2.16^b^	38.80 ± 0.42^b^	106.85 ± 2.23^b^
180°C	10 min	5.14 ± 0.08^b^	19.44 ± 0.68^c^	15.24 ± 0.42^h^	88.19 ± 0.16^e^
	20 min	5.45 ± 0.19^ab^	4.55 ± 1.78^e^	19.76 ± 1.10^g^	96.99 ± 2.93^d^
	30 min	5.41 ± 0.13^ab^	1.84 ± 0.68^e^	36.68 ± 0.42^c^	91.19 ± 3.23^e^

Values (mean ± SD, *n* = 3) in the same column followed by a different letter are significantly different (*p* < 0.05).

The results of free radicals scavenging capacity and OSI indicated that the roasting treatment can strengthen the antioxidant capacity of walnut oil. The research results of Aleksander et al. on the antioxidant capacity of rapeseed oil prepared after roasting are consistent with this study (40). The antioxidant activity of Sacha Inchi oil is also improved by roasting Sacha Inchi (*Plukenetia Volubilis L.*) seeds (41). Plants contain many phenolic compounds with antioxidant capacity in the combined form (42). The roasting treatment may destroy these phenolic compounds in the form of covalent bonding, and release the phenolic compounds in free form. The marked enhancement in the antioxidant capacity of the oil after roasting treatment may be attributed to the degradation of some heat-insensitive antioxidant components to produce heat-resistant antioxidant components and the formation of some antioxidants with potential antioxidant activity by Maillard reaction (17, 43).

### 3.4. PCA and HCA

**FIGURE 2:**
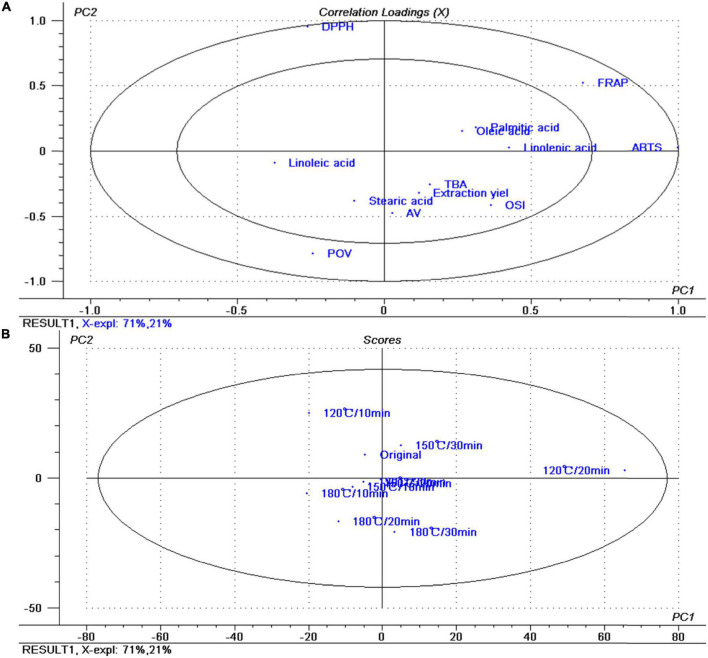
Principal component analysis of the data. **(A)** Loadings plot. **(B)** Scores plot.

Principal component analysis aims to use mathematical dimensionality reduction methods to transform multiple variables into a small number of new variables using orthogonal changes, thereby using new variables to reflect the main characteristics of things in a smaller dimension. Thirteen variables were employed for statistically evaluating including extraction yield, physicochemical characteristics, and antioxidant capacity (Figure 2A).

We note that the first two component scores consider 92% (component 1 = 71% and component 2 = 21%) of the total variation. Component 1 exhibits positive loading mainly with ABTS, FRAP, linolenic acid, OSI, palmitic acid, and negative loading with linoleic acid. The main contributors to principal component 2 are DPPH, POV, FRAP, AV, OSI, stearic acid, and extraction yield. The figure showed that the sample roasted at 120°C for 20 min can be clearly distinguished from other samples (Figure 2B). According to the above research, considering that the samples roasted at 120°C for 20 min have better antioxidant capacity, this roasting treatment is a suitable pretreatment roasting condition for pressing walnut oil.

**FIGURE 3:**
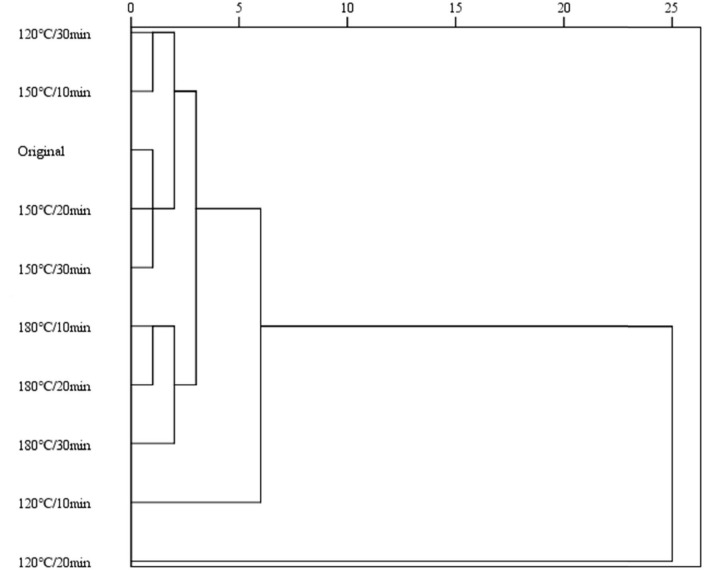
Hierarchical cluster analysis of walnut oil.

Hierarchical cluster analysis is a type of clustering algorithm. It uses Euclidean distance to calculate the distance (similarity) between different data points and sequentially combines the two closest data points to generate a hierarchical nested clustering tree (Figure 3). The smaller the distance between data points, the higher the similarity. These results show that walnut oil samples have a clear tendency to aggregate. The tree structure of the cluster analysis was divided into two main parts when the 10-distance threshold was chosen. The sample roasted at 120°C for 20 min can be clearly distinguished from other samples. In summary, we can conclude that the sample roasted at 120°C for 20 min is the better processing condition.

### 3.5. Correlations between indicators of walnut oils

**FIGURE 4:**
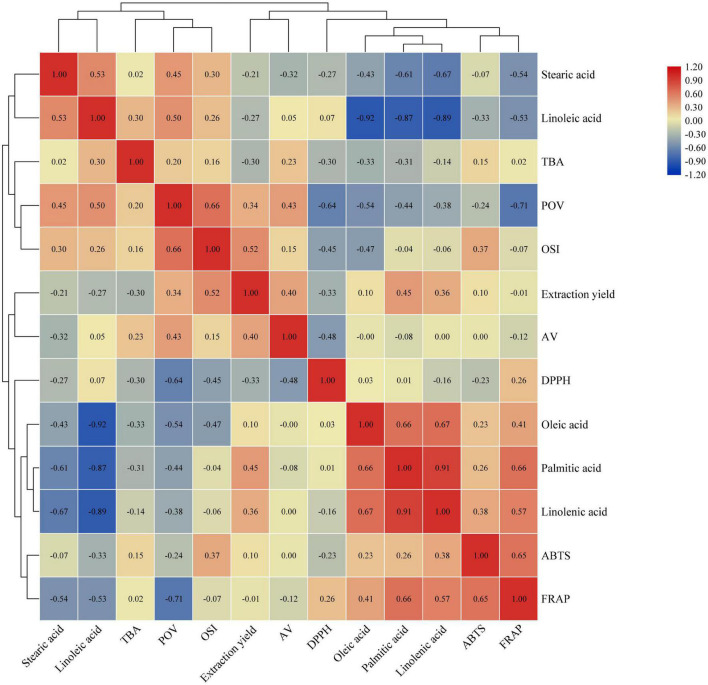
Correlation heatmap between walnut oil indicators.

Heatmap is a common visualization method in scientific research papers. The correlation of samples is often displayed in the form of correlation heatmap. In summary, any value that characterizes the correlation can be plotted using a correlation heatmap. The depth of color in the correlation heatmap can clearly show the strength of the correlation among indicators (Figure 4). The red color represents a positive correlation, blue color represents a negative correlation. Palmitic acid had a significant positive correlation with linolenic acid (*r* = 0.905, *p* < 0.01), oleic acid (*r* = 0.657, *p* < 0.05) and FRAP (*r* = 0.657, *p* < 0.05), and a significant negative correlation with linoleic acid (*r* = –0.871, *p* < 0.01). POV showed a significant positive correlation with OSI (*r* = 0.663, *p* < 0.05), and a significant negative correlation with DPPH (*r* = –0.641, *p* < 0.05) and FRAP (*r* = –0.709, *p* < 0.05). Linolenic acid was significant negative correlated with stearic acid (*r* = –0.673, *p* < 0.05) and linoleic acid (*r* = –0.894, *p* < 0.01), and positively correlated with oleic acid (*r* = 0.672, *p* < 0.05). Linoleic acid displayed a significant negative correlation on oleic acid (*r* = –0.922, *p* < 0.01). FRAP exhibited a significant positive correlation on ABTS (*r* = 0.650, *p* < 0.05). There is no significant relationship between the rest. At the same time, these indicators can be divided into two categories through cluster analysis. The first category includes TBA, stearic acid, linoleic acid, POV, and OSI. The other category includes DPPH, extraction yield, AV, palmitic acid, oleic acid, linolenic acid, ABTS, and FRAP.

### 3.6. HS-GC-IMS analysis

**FIGURE 5:**
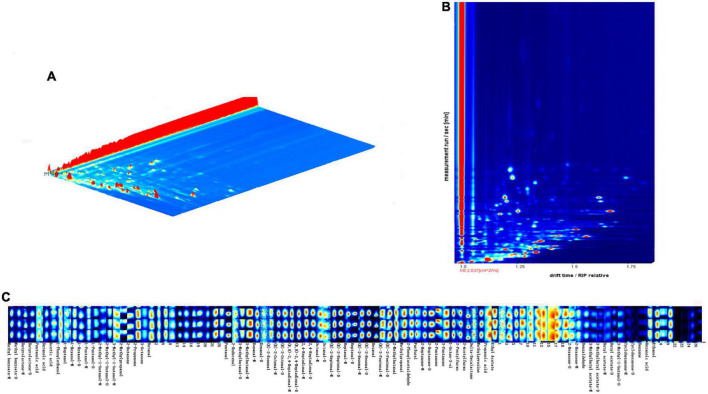
3D topography **(A)**, 2D-topographic plots **(B)** and fingerprinting of VOCs **(C)** of walnut oil under optimal roasting treatment (120°C, 20 min).

Volatiles of walnut oil (120°C, 20 min) were plotted in 3D topography (Figure 5A). The X, Y, and Z axis in 3D spectrum correspond to ion drift identification time, gas chromatography retention time and quantitative peak height, respectively. Figure 5B shows the gas-phase ion mobility profiles of volatile compounds from walnut oil. The vertical coordinate is the retention time (s) of the compound, and the horizontal coordinate is the ion migration time (ms). Each point on the spectrum represents a volatile compound, with red representing high concentration and white representing low concentration, and the darker the color indicates the higher concentration of the volatile compound. The results of the qualitative analysis of volatile compounds using the NIST database and IMS database integrated with the software are shown in Supplementary Table. A total of 73 volatile compounds were detected, including 30 aldehydes, 13 alcohols, 11 ketones, 10 esters, 5 acids, 2 oxygen-containing heterocycles, 1 nitrogen-containing heterocycle and 1 other compound. Figure 5C shows the fingerprint profile, each row of the figure represents the peak intensity of all volatile compounds in the walnut oil sample, and each column represents the peak intensity of a volatile compound in the walnut oil sample. Dimer or polymer formation in the ionized region is associated with high proton affinity of the volatile compound and results in a varying drift time in contrast to the monomeric form, so that some compounds can produce two peak signals, including dimer (D) and monomer (M), as observed in GC-IMS (44).

Aldehydes are mainly derived from the cleavage of free radicals of lipid molecules. The peroxides generated by auto-oxidation of lipids decompose to form alkoxy radicals and hydroxyl radicals, which further cleave to form volatile organic compounds such as aldehydes, alkenes and alcohols (45, 46). Aldehydes have aromatic characteristics such as clear, fruity, and fatty aromas, and their detection concentration is high and the aroma threshold is low, which is the main aroma presenting substance of walnut oil. Among them, (*E*)-2-heptenal, (*E*)-2-pentenal and hexanal were the aldehydes with the highest peak intensity in the pretreated walnut oil at 120°C, 20 min. (*E*)-2-heptenal had a green, herbal or cucumber aroma and was a high-concentration volatile compound in cooked white quinoa samples (47). (*E)*-2-Pentenal was considered as a potential marker for linolenic acid-based vegetable oils (48). Hexanal was derived from 13-hydroperoxides formed by the autoxidation of linoleic acid and was directly related to the formation of off-flavors from the oxidation of oils and fats (49, 50). Ketones are produced through lipid degradation, Maillard reaction and interaction between these two reactions. In addition, ketones have a comparatively high-odor threshold, contributing to a minor flavor impact on food (51). 2-Acetone was the volatile compound with the highest peak intensity in walnut oil (5,480). The ester and aldehyde with the highest peak areas in walnut oil were ethyl acetate (pineapple flavor) and ethanol (sweetness), respectively, which were considered potential markers of yogurt flavor quality (52).

## 4. Conclusion

In this work, we investigated the effects of roasting treatment on the physicochemical properties, antioxidant capacity and correlation between indicators of walnut oil, and further measured the flavor substances of walnut oil under optimal roasting treatment. Linoleic acid (62.31% of the crude oil) and oleic acid (12.40% of crude oil) were the predominant fatty acids in the oil extracted from walnut kernels without roasted. Moderate roasting could increase the content of unsaturated fatty acids (oleic acid and linoleic acid) and saturated fatty acids (palmitic acid), especially the sample roasted 120°C for 30 min. As the roasting temperature and duration increases, it caused a statistically significant increase in POV relative to the control sample. PCA and HCA could distinguish walnut oil from different roasting treatments, and the result showed that roasting at 120°C for 20 min was a suitable pretreatment roasting condition for pressing walnut oil. There were significant influences among indicators. POV had a significant positive correlation with OSI (*r* = 0.633, *p* < 0.05), and a significant negative correlation with DPPH (*r* = –0.641, *p* < 0.05) and FRAP (*r* = –0.709, *p* < 0.05). Palmitic acid had a significant positive correlation on FRAP (*r* = 0.657, *p* < 0.05). Antioxidant capacity (OSI, DPPH, and FRAP) could be guided by the correlation between POV and palmitic acid. The study also rapidly identified VOCs in walnut oil obtained under suitable pretreatment roasting conditions. Aldehydes with clear, fruity, and fatty aromas were considered as the main aroma-presenting substances of walnut oil.

## Data availability statement

The original contributions presented in this study are included in the article/Supplementary material, further inquiries can be directed to the corresponding authors.

## Author contributions

HL: conceptualization, methodology, formal analysis, and writing—original draft. JH: formal analysis, writing—original draft, and writing—review and editing. ZZ and JT: writing—review and editing, resources, and supervision. XF: supervision and writing—review and editing. YZ: writing—review and editing. CW and WL: funding acquisition, resources, and supervision. All authors contributed to the article and approved the submitted version.

## References

[1] Aradhya M Potter D Gao F Simon C. Molecular phylogeny of Juglans (*Juglandaceae)*: a biogeographic perspective. *Tree Genet Genomes.* (2007) 3:363–78. 10.1007/s11295-006-0078-5

[2] Wang P Zhong L Yanga H Zhu F Hou X Wu C Comparative analysis of antioxidant activities between dried and fresh walnut kernels by metabolomic approaches. *LWT Food Sci Technol.* (2022) 155:112875. 10.1016/j.lwt.2021.112875

[3] Zhou Y Fan W Chu F Pei D. Improvement of the flavor and oxidative stability of walnut oil by microwave pretreatment. *J Am Oil Chem Soc.* (2016) 93:1563–72. 10.1007/s11746-016-2891-9

[4] Gharibzahedi S Mousavi S Hamedi M Khodaiyan F. Determination and characterization of kernel biochemical composition and functional compounds of Persian walnut oil. *J Food Sci Technol.* (2014) 51:34–42. 10.1007/s13197-011-0481-2 24426045 PMC3857422

[5] Pereira J Oliveira I Sousa A Ferreira I Bento A Estevinho L. Bioactive properties and chemical composition of six walnut (*Juglans regia L*.) cultivars. *Food Chem Toxicol.* (2008) 46:2103–11. 10.1016/j.fct.2008.02.002 18334279

[6] Rébufa C Artaud J Le Dréau Y. Walnut (*Juglans regia L*.) oil chemical composition depending on variety, locality, extraction process and storage conditions: a comprehensive review. *J Food Compos Anal.* (2022) 110:104534. 10.1016/j.jfca.2022.104534

[7] Mildner-Szkudlarz S Różańska M Siger A Kowalczewski P Rudzińska M. Changes in chemical composition and oxidative stability of cold-pressed oils obtained from by-product roasted berry seeds. *LWT Food Sci Technol.* (2019) 111:541–7. 10.1016/j.lwt.2019.05.080

[8] Rękas A Wroniak M Rusinek R. Influence of roasting pretreatment on high-oleic rapeseed oil quality evaluated by analytical and sensory approaches. *Int J Food Sci Technol.* (2015) 50:2208–14. 10.1111/ijfs.12884

[9] Mikołajczak N Tańska M Ogrodowska D. Phenolic compounds in plant oils: a review of composition, analytical methods, and effect on oxidative stability. *Trends Food Sci Technol.* (2021) 113:110–38. 10.1016/j.tifs.2021.04.046

[10] Siger A Michalak M. The long-term storage of cold-pressed oil from roasted rapeseed: effects on antioxidant activity and levels of canolol and tocopherols. *Eur J Lipid Sci Technol.* (2015) 118:1030–41. 10.1002/ejlt.201500183

[11] Potoènik T Rak Cizej M Košir I. Influence of seed roasting on pumpkin seed oil tocopherols, phenolics and antiradical activity. *J Food Compos Anal.* (2018) 69:7–12. 10.1016/j.jfca.2018.01.020

[12] EmÝR D AydenÝZ B Yilmaz E. Effects of roasting and enzyme pretreatments on yield and quality of cold-pressed poppy seed oils. *Turk J Agric For.* (2015) 39:260–71. 10.1002/jsfa.1904

[13] Parker J. Thermal generation or aroma. In: Parker JK Elmore JS Methven L editors. *Flavour Development, Analysis and Perception in Food and Beverages.* Sawston: Woodhead publishing (2015). p. 151–85. 10.1016/B978-1-78242-103-0.00008-4

[14] Chang S Alasalvar C Bolling B Shahidi F. Nuts and their co-products: the impact of processing (roasting) on phenolics, bioavailability, and health benefits – A comprehensive review. *J Funct Foods.* (2016) 26:88–122. 10.1016/j.jff.2016.06.029

[15] Vinson J Cai Y. Nuts, especially walnuts, have both antioxidant quantity and efficacy and exhibit significant potential health benefits. *Food Funct.* (2012) 3:134–40. 10.1039/c2fo10152a 22187094

[16] Chandrasekara N Shahidi F. Effect of roasting on phenolic content and antioxidant activities of whole cashew nuts, kernels, and testa. *J Agric Food Chem.* (2011) 59:5006–14. 10.1021/jf2000772 21438525

[17] Dewanto V Wu X Adom K Liu R. Thermal processing enhances the nutritional value of tomatoes by increasing total antioxidant activity. *J Agric Food Chem.* (2002) 50:3010–4. 10.1021/jf0115589 11982434

[18] Gao P Liu R Jin Q Wang X. Effects of processing methods on the chemical composition and antioxidant capacity of walnut (*Juglans regia L*.) oil. *LWT Food Sci Technol.* (2021) 135:109958. 10.1016/j.lwt.2020.109958

[19] Belviso S Dal Bello B Giacosa S Bertolino M Ghirardello D Giordano M Chemical, mechanical and sensory monitoring of hot air- and infrared-roasted hazelnuts (*Corylus avellana L*.) during nine months of storage. *Food Chem.* (2017) 217:398–408. 10.1016/j.foodchem.2016.08.103 27664651

[20] Lin J Liu S Hu C Shyu Y Hsu C Yang D. Effects of roasting temperature and duration on fatty acid composition, phenolic composition, Maillard reaction degree and antioxidant attribute of almond (*Prunus dulcis*) kernel. *Food Chem.* (2016) 190:520–8. 10.1016/j.foodchem.2015.06.004 26213005

[21] Angerosa F Servili M Selvaggini R Taticchi A Esposto S Montedoro G. Volatile compounds in virgin olive oil: occurrence and their relationship with the quality. *J Chromatogr A.* (2004) 1054:17–31. 10.1016/s0021-9673(04)01298-1 15553127

[22] Kandylis P Vekiari A Kanellaki M Grati Kamoun N Msallem M Kourkoutas Y. Comparative study of extra virgin olive oil flavor profile of Koroneiki variety (*Olea europaea* var. *Microcarpa alba*) cultivated in Greece and Tunisia during one period of harvesting. *LWT Food Sci Technol.* (2011) 44:1333–41. 10.1016/j.lwt.2010.12.021

[23] Yu P Yang Y Zhou Q Jia X Zheng C Zhou Q Identification of volatile sulfur-containing compounds and the precursor of dimethyl sulfide in cold-pressed rapeseed oil by GC–SCD and UPLC–MS/MS. *Food Chem.* (2022) 367:130741. 10.1016/j.foodchem.2021.130741 34399272

[24] Wei C Xi W Nie X Liu W Wang Q Yang B Aroma characterization of flaxseed oils using headspace solid-phase microextraction and gas chromatography-olfactometry. *Eur J Lipid Sci Technol.* (2013) 115:1032–42. 10.1002/ejlt.201200397

[25] Cavanna D Zanardi S Dall’Asta C Suman M. Ion mobility spectrometry coupled to gas chromatography: a rapid tool to assess eggs freshness. *Food Chem.* (2019) 271:691–6. 10.1016/j.foodchem.2018.07.204 30236732

[26] Babis J Sperline R Knight A Jones D Gresham C Denton M. Performance evaluation of a miniature ion mobility spectrometer drift cell for application in hand-held explosives detection ion mobility spectrometers. *Anal Bioanal Chem.* (2009) 395:411–9. 10.1007/s00216-009-2818-5 19424683

[27] Li Q Li R Cao G Wu X Yang G Cai B Direct differentiation of herbal medicine for volatile components by a multicapillary column with ion mobility spectrometry method. *J Sep Sci.* (2015) 38:3205–8. 10.1002/jssc.201500402 26152210

[28] Arnanthigo Y Anttalainen O Safaei Z Sillanpää M. Sniff-testing for indoor air contaminants from new buildings environment detecting by aspiration-type ion mobility spectrometry. *Int J Ion Mobility Spectrom.* (2016) 19:15–30. 10.1007/s12127-016-0189-0

[29] Mochalski P Wiesenhofer H Allers M Zimmermann S Guntner A Pineau N Monitoring of selected skin- and breath-borne volatile organic compounds emitted from the human body using gas chromatography ion mobility spectrometry (GC-IMS). *J Chromatogr B Analyt Technol Biomed Life Sci.* (2018) 1076:29–34. 10.1016/j.jchromb.2018.01.013 29396365

[30] Pu D Zhang H Zhang Y Sun B Ren F Chen H Characterization of the aroma release and perception of white bread during oral processing by gas chromatography-ion mobility spectrometry and temporal dominance of sensations analysis. *Food Res Int.* (2019) 123:612–22. 10.1016/j.foodres.2019.05.016 31285010

[31] Li M Yang R Zhang H Wang S Chen D Lin S. Development of a flavor fingerprint by HS-GC-IMS with PCA for volatile compounds of *Tricholoma matsutake* singer. *Food Chem.* (2019) 290:32–9. 10.1016/j.foodchem.2019.03.124 31000053

[32] Arroyo-Manzanares N Martin-Gomez A Jurado-Campos N Garrido-Delgado R Arce C Arce L. Target vs spectral fingerprint data analysis of Iberian ham samples for avoiding labelling fraud using headspace - gas chromatography-ion mobility spectrometry. *Food Chem.* (2018) 246:65–73. 10.1016/j.foodchem.2017.11.008 29291880

[33] Mackey P Whitaker M. Diabetes mellitus and hyperglycemia management in the hospitalized patient. *J Nurse Pract.* (2015) 11:531–7. 10.1016/j.nurpra.2015.02.016

[34] Liu S Lin J Wang C Chen H Yang D. Antioxidant properties of various solvent extracts from lychee (*Litchi chinenesis Sonn.*) flowers. *Food Chem.* (2009) 114:577–81. 10.1016/j.foodchem.2008.09.088

[35] Gao P Jin J Liu R Jin Q Wang X. Chemical compositions of Walnut (*Juglans regiaL*.) oils from different cultivated regions in China. *J Am Oil Chem Soc.* (2018) 95:825–34. 10.1002/aocs.12097

[36] Re R Pellegrini N Proteggente A Pannala A Yang M Rice-Evans C. Antioxidant activity applying an improved ABTS radical cation decolorization assay. *Free Radical Biol Med.* (1999) 26:1231–7. 10.1016/S0891-5849(98)00315-3 10381194

[37] Vaidya B Eun J. Effect of roasting on oxidative and tocopherol stability of walnut oil during storage in the dark. *Eur J Lipid Sci Technol.* (2013) 115:348–55. 10.1002/ejlt.201200288

[38] Zhao T Hong S Lee J Lee J Kim I. Impact of roasting on the chemical composition and oxidative stability of *Perilla* oil. *J Food Sci.* (2012) 77:C1273–8. 10.1111/j.1750-3841.2012.02981.x 23140339

[39] Gunstone F. *Edible Oil and Fat Products: Chemistry, Properties, and Health Effects. Fereidoon Shahidi. Bailey’s Industrial Oil and Fat Products.* (Vol. 1), New York, NY: John Wiley & Sons (2005).

[40] Aleksander Siger M Lampart-Szczapa E. The content and antioxidant activity of phenolic compounds in cold-pressed plant oils. *J Food Lipids.* (2007) 15:137–49. 10.1111/j.1745-4522.2007.00107.x

[41] Cisneros F Paredes D Arana A Cisneros-Zevallos L. Chemical composition, oxidative stability and antioxidant capacity of oil extracted from roasted seeds of Sacha-inchi (*Plukenetia volubilis L*.). *J Agric Food Chem.* (2014) 62:5191–7. 10.1021/jf500936j 24823227

[42] Lee S Kim J-H Jeong S-M Kim D Ha J Nam K Effect of far-infrared radiation on the antioxidant activity of rice hulls. *J Agric Food Chem.* (2003) 51:4400–3. 10.1021/JFO300285 12848517

[43] Nicoli M Anese M Parpinel M. Influence of processing on the antioxidant properties of fruit and vegetables. *Trends Food Sci Technol.* (1999) 10:94–100. (99)00023-0 10.1016/s0924-2244

[44] Chen Y Li P Liao L Qin Y Jiang L Liu Y. Characteristic fingerprints and volatile flavor compound variations in Liuyang Douchi during fermentation via HS-GC-IMS and HS-SPME-GC-MS. *Food Chem.* (2021) 361:130055. 10.1016/j.foodchem.2021.130055 34023693

[45] Sun X Wang Y Li H Zhou J Han J Wei C. Changes in the volatile profile, fatty acid composition and oxidative stability of flaxseed oil during heating at different temperatures. *LWT Food Sci Technol.* (2021) 151:112137. 10.1016/j.lwt.2021.112137

[46] Yang Y Yu P Sun J Jia Y Wan C Zhou Q Investigation of volatile thiol contributions to rapeseed oil by odor active value measurement and perceptual interactions. *Food Chem.* (2022) 373:131607. 10.1016/j.foodchem.2021.131607 34819247

[47] Yang X Zhu K Guo H Geng Y Lv W Wang S Characterization of volatile compounds in differently coloured *Chenopodium quinoa* seeds before and after cooking by headspace-gas chromatography-ion mobility spectrometry. *Food Chem.* (2021) 348:129086. 10.1016/j.foodchem.2021.129086 33508608

[48] Hu Q Zhang J He L Xing R Yu N Chen Y. New insight into the evolution of volatile profiles in four vegetable oils with different saturations during thermal processing by integrated volatolomics and lipidomics analysis. *Food Chem.* (2022) 403:134342. 10.1016/j.foodchem.2022.134342 36162262

[49] Fullana A Carbonell-Barrachina AA Sidhu S. Volatile aldehyde emissions from heated cooking oils. *J Sci Food Agric.* (2004) 84:2015–21.

[50] Xu Y Bi S Zhou Q Dai Y Zhou Q Liu Y Identification of aroma active compounds in walnut oil by monolithic material adsorption extraction of RSC18 combined with gas chromatography-olfactory-mass spectrometry. *Food Chem.* (2023) 402:134303. 10.1016/j.foodchem.2022.134303 36152552

[51] Wang Y Tang X Luan J Zhu W Xu Y Yi S Effects of ultrasound pretreatment at different powers on flavor characteristics of enzymatic hydrolysates of cod (*Gadus macrocephalus*) head. *Food Res Int.* (2022) 159:111612. 10.1016/j.foodres.2022.111612 35940806

[52] Zhang Y Lin Q Zhong H Zeng Y. Identification and source analysis of volatile flavor compounds in paper packaged yogurt by headspace solid-phase microextraction-gas chromatography-mass spectrometry. *Food Packag Shelf Life.* (2022) 34:100947. 10.1016/j.fpsl.2022.100947

