# Supplementary material for: Roasting treatments affect oil extraction rate, fatty acids, oxidative stability, antioxidant activity, and flavor of walnut oil
Source: Front Nutr. 2023 Jan 4;9:1077081. doi: 10.3389/fnut.2022.1077081 (PMC9846541; doi:10.3389/fnut.2022.1077081)
Supplement: Supplementary file 1 [file Table_1.docx]

**Supplementary Table**

Identification of volatile compound of walnut oil by GC-IMS

| **Count** | **Compound** | **CAS** | **Formula** | **MW** | **RI** | **Rt [sec]** | **Dt [RIP rel]** | **Signal Intensity** |
| --- | --- | --- | --- | --- | --- | --- | --- | --- |
|  |  |  |  |  |  |  |  | **walnut oil（120℃, 20 min）** |
| 1 | Delta-Decalactone | C705862 | C10H18O2 | 170.3 | 1550.3 | 1156.206 | 1.426 | 503±4 |
| 2 | 2-Undecenal | C2463776 | C11H20O | 168.3 | 1392.3 | 924.141 | 1.488 | 684±24 |
| 3 | (E)-2-Nonenal | C18829566 | C9H16O | 140.2 | 1181.9 | 615.082 | 1.412 | 223±15 |
| 4 | Nonanal-M | C124196 | C9H18O | 142.2 | 1104.1 | 500.83 | 1.479 | 617±28 |
| 5 | Nonanal-D | C124196 | C9H18O | 142.2 | 1104.1 | 500.83 | 1.947 | 89±2 |
| 6 | Methyl benzoate-M | C93583 | C8H8O2 | 136.1 | 1088.8 | 478.342 | 1.219 | 1991±68 |
| 7 | Methyl benzoate-D | C93583 | C8H8O2 | 136.1 | 1088.8 | 478.342 | 1.605 | 222±7 |
| 8 | (E)-2-Octenal-M | C2548870 | C8H14O | 126.2 | 1052.8 | 425.463 | 1.334 | 979±5 |
| 9 | (E)-2-Octenal-D | C2548870 | C8H14O | 126.2 | 1052.8 | 425.463 | 1.822 | 191±10 |
| 10 | (E,E)-2,4-Heptadienal-M | C4313035 | C7H10O | 110.2 | 1010.7 | 363.631 | 1.199 | 1873±18 |
| 11 | (E,E)-2,4-Heptadienal-D | C4313035 | C7H10O | 110.2 | 1010.7 | 363.631 | 1.625 | 1472±8 |
| 12 | 2,4-Heptadienal-M | C5910850 | C7H10O | 110.2 | 999.5 | 347.197 | 1.204 | 1019±6 |
| 13 | 2,4-Heptadienal-D | C5910850 | C7H10O | 110.2 | 1000.1 | 348.136 | 1.631 | 747±11 |
| 14 | Octanal-M | C124130 | C8H16O | 128.2 | 1004.3 | 354.24 | 1.411 | 204±13 |
| 15 | Octanal-D | C124130 | C8H16O | 128.2 | 1004.3 | 354.24 | 1.828 | 41±7 |
| 16 | 1-Octen-3-ol | C3391864 | C8H16O | 128.2 | 981.6 | 330.763 | 1.157 | 665±12 |
| 17 | (E)-2-Heptenal-M | C18829555 | C7H12O | 112.2 | 953.2 | 307.727 | 1.256 | 1600±11 |
| 18 | (E)-2-Heptenal-D | C18829555 | C7H12O | 112.2 | 952.7 | 307.355 | 1.669 | 2983±6 |
| 19 | Pentanoic acid | C109524 | C5H10O2 | 102.1 | 910.8 | 273.454 | 1.234 | 136±9 |
| 20 | Heptanal-M | C111717 | C7H14O | 114.2 | 894.7 | 260.415 | 1.338 | 585±17 |
| 21 | Heptanal-D | C111717 | C7H14O | 114.2 | 895.6 | 261.16 | 1.698 | 289±9 |
| 22 | 2-Heptanone-M | C110430 | C7H14O | 114.2 | 884.3 | 254.082 | 1.264 | 382±6 |
| 23 | 2-Heptanone-D | C110430 | C7H14O | 114.2 | 883.6 | 253.71 | 1.636 | 298±2 |
| 24 | (E)-2-Hexenal-M | C6728263 | C6H10O | 98.1 | 839.6 | 229.896 | 1.182 | 686±6 |
| 25 | (E)-2-Hexenal-D | C6728263 | C6H10O | 98.1 | 840.1 | 230.141 | 1.516 | 1788±9 |
| 26 | Hexanal | C66251 | C6H12O | 100.2 | 787.3 | 201.537 | 1.563 | 2258±11 |
| 27 | 1-Pentanol-M | C71410 | C5H12O | 88.1 | 757.4 | 188.58 | 1.255 | 524±2 |
| 28 | 1-Pentanol-D | C71410 | C5H12O | 88.1 | 756.2 | 188.091 | 1.52 | 629±6 |
| 29 | (E)-2-Pentenal-M | C1576870 | C5H8O | 84.1 | 743.4 | 182.712 | 1.107 | 406±12 |
| 30 | (E)-2-Pentenal-D | C1576870 | C5H8O | 84.1 | 742.3 | 182.223 | 1.36 | 2625±21 |
| 31 | Pentanal | C110623 | C5H10O | 86.1 | 693.0 | 161.443 | 1.423 | 980±10 |
| 32 | 3-Methylbutanal-M | C590863 | C5H10O | 86.1 | 645.4 | 146.577 | 1.2 | 1932±13 |
| 33 | 2-Pentanone | C107879 | C5H10O | 86.1 | 682.3 | 157.691 | 1.373 | 244±1 |
| 34 | 2-Butanone | C78933 | C4H8O | 72.1 | 593.5 | 130.948 | 1.246 | 1080±22 |
| 35 | 2-Propanone | C67641 | C3H6O | 58.1 | 504.2 | 104.031 | 1.116 | 5480±109 |
| 36 | 2-Phenylacetaldehyde | C122781 | C8H8O | 120.2 | 1026.4 | 386.822 | 1.263 | 195±2 |
| 37 | 2-Pentylfuran | C3777693 | C9H14O | 138.2 | 993.9 | 340.74 | 1.252 | 270±3 |
| 38 | Benzaldehyde | C100527 | C7H6O | 106.1 | 958.6 | 312.12 | 1.151 | 168±6 |
| 39 | 2-Acetylfuran | C1192627 | C6H6O2 | 110.1 | 906.5 | 269.919 | 1.119 | 725±11 |
| 40 | 3-Methylbutyl acetate-M | C123922 | C7H14O2 | 130.2 | 867.4 | 244.949 | 1.308 | 55±1 |
| 41 | 3-Methylbutyl acetate-D | C123922 | C7H14O2 | 130.2 | 866.9 | 244.637 | 1.75 | 57±1 |
| 42 | 1-Hexanol-M | C111273 | C6H14O | 102.2 | 861.1 | 241.518 | 1.329 | 796±13 |

**Supplementary Table** *(continued)*

| Count | Compound | CAS | Formula | MW | RI | Rt [sec] | Dt [RIP rel] | Signal Intensity |
| --- | --- | --- | --- | --- | --- | --- | --- | --- |
|  |  |  |  |  |  |  |  | walnut oil（120℃，20min） |
| 43 | 1-Hexanol-D | C111273 | C6H14O | 102.2 | 862.2 | 242.141 | 1.647 | 591±3 |
| 44 | Methylpyrazine | C109080 | C5H6N2 | 94.1 | 820.2 | 219.373 | 1.084 | 344±1 |
| 45 | Furfural | C98011 | C5H4O2 | 96.1 | 821.3 | 219.996 | 1.335 | 369±5 |
| 46 | Butyl acetate-M | C123864 | C6H12O2 | 116.2 | 801.7 | 209.392 | 1.236 | 76±3 |
| 47 | Butyl acetate-D | C123864 | C6H12O2 | 116.2 | 799.4 | 208.144 | 1.62 | 61±4 |
| 48 | 2-Hexanone-M | C591786 | C6H12O | 100.2 | 775.7 | 196.292 | 1.194 | 91±3 |
| 49 | 2-Hexanone-D | C591786 | C6H12O | 100.2 | 776.4 | 196.604 | 1.501 | 55±4 |
| 50 | 3-Methyl-1-butanol-M | C123513 | C5H12O | 88.1 | 723.1 | 174.147 | 1.248 | 90±4 |
| 51 | 3-Methyl-1-butanol-D | C123513 | C5H12O | 88.1 | 723.1 | 174.147 | 1.495 | 50±2 |
| 52 | 3-Pentanone | C96220 | C5H10O | 86.1 | 679.8 | 156.937 | 1.343 | 557±24 |
| 53 | 2-Methylbutanal | C96173 | C5H10O | 86.1 | 661.4 | 151.407 | 1.404 | 1158±13 |
| 54 | 3-Methylbutanal-D | C590863 | C5H10O | 86.1 | 643.1 | 145.877 | 1.411 | 434±8 |
| 55 | Ethyl Acetate | C141786 | C4H8O2 | 88.1 | 614.9 | 137.398 | 1.337 | 3988±15 |
| 56 | Hexanoic acid | C142621 | C6H12O2 | 116.2 | 1023.5 | 382.462 | 1.299 | 611±18 |
| 57 | Propanoic acid | C79094 | C3H6O2 | 74.1 | 676.4 | 155.941 | 1.265 | 166±1 |
| 58 | 2-Methylpropanol | C78831 | C4H10O | 74.1 | 622.2 | 139.61 | 1.172 | 172±4 |
| 59 | Ethanol | C64175 | C2H6O | 46.1 | 474.8 | 95.184 | 1.046 | 2210±32 |
| 60 | Acetic acid | C64197 | C2H4O2 | 60.1 | 575.0 | 125.387 | 1.052 | 711±23 |
| 61 | 1-Heptanol | C111706 | C7H16O | 116.2 | 974.7 | 325.212 | 1.402 | 194±5 |
| 62 | Butyrolactone-M | C96480 | C4H6O2 | 86.1 | 911.9 | 274.326 | 1.082 | 561±12 |
| 63 | Butyrolactone-D | C96480 | C4H6O2 | 86.1 | 910.5 | 273.188 | 1.302 | 175±7 |
| 64 | Butanal | C123728 | C4H8O | 72.1 | 598.9 | 132.565 | 1.29 | 990±13 |
| 65 | 1-Phenylethanol | C98851 | C8H10O | 122.2 | 1050.3 | 421.793 | 1.192 | 149±7 |
| 66 | 3-Octanone | C106683 | C8H16O | 128.2 | 988.5 | 336.384 | 1.306 | 135±5 |
| 67 | 2-Methyl-1-butanol-M | C137326 | C5H12O | 88.1 | 745.3 | 183.486 | 1.221 | 65±1 |
| 68 | 2-Methyl-1-butanol-D | C137326 | C5H12O | 88.1 | 744.8 | 183.294 | 1.488 | 274±6 |
| 69 | Butanoic acid | C107926 | C4H8O2 | 88.1 | 817.4 | 217.87 | 1.161 | 229±2 |
| 70 | Methylpropanal | C78842 | C4H8O | 72.1 | 553.6 | 118.927 | 1.287 | 2308±24 |
| 71 | Cyclohexanone-M | C108941 | C6H10O | 98.1 | 890.4 | 257.384 | 1.157 | 168±2 |
| 72 | Cyclohexanone-D | C108941 | C6H10O | 98.1 | 890.0 | 257.167 | 1.458 | 16±2 |
| 73 | Benzene | C71432 | C6H6 | 78.1 | 664.0 | 152.205 | 0.988 | 106±3 |
